# Circulation of *Trypanosoma cruzi* TcI in wild populations and with a tendency toward domiciliation of *Panstrongylus geniculatus* (Latreille, 1811) (Hemiptera; Reduviidae) in the department of Atlántico, Colombia

**DOI:** 10.17843/rpmesp.2026.431.15385

**Published:** 2026-03-06

**Authors:** Yeisson Cera-Vallejo, Claudia Romero-Vivas, Andrew K. Falconar, Roberto García-Alzate, Daisy Lozano-Arias

**Affiliations:** 1 Tropical Diseases Research Group, Universidad del Norte, Puerto Colombia, Atlántico, Colombia.; 2 Grupo de investigación en Ciencias Básicas y Clínicas GIBAC, Facultad de Ciencias de la Salud, Fundación Universitaria San Martín-Puerto Colombia, Atlántico, Colombia.; 3 Basic and Clinical Sciences Research Group GIBAC, Faculty of Health Sciences, Fundación Universitaria San Martín-Puerto Colombia, Atlántico, Colombia.

**Keywords:** Trypanosoma cruzi, genotype, Panstrongylus

## Abstract

The TcI genotype of *Trypanosoma cruzi* was identified in wild populations and those with a tendency toward domiciliation of *Panstrongylus geniculatus* in the department of Atlántico, Colombia. Through active searching and light traps in rural and urban areas of three municipalities, 33 specimens of *P. geniculatus* were captured: Usiacurí (2), Piojó (2), and Tubará (29), the latter collected in five households. DNA from the digestive content was analyzed by PCR using molecular markers (DNAk, Mini-Exon, 18S, DNAr). Infection by *T. cruzi* was evidenced in 75% of the specimens (25/33), all from Tubará, with exclusive identification of the TcI genotype. Additionally, entomological indices were calculated, with infestation, colonization, and natural infection indices of 60%, 33%, and 86%, respectively. These findings constitute the first report of *T. cruzi* TcI in the department of Atlántico and the first record of *P. geniculatus* in Usiacurí, highlighting a potential risk to Colombian public health.

## INTRODUCTION

Chagas disease (CD) is a zoonosis, primarily vector-borne, that affects vulnerable communities in 21 Latin American countries. It is estimated that around seven million people are infected, with 30,000 cases and 12,000 deaths annually ^1^. Its etiological agent is *Trypanosoma (Schizotrypanum) cruzi* Chagas 1909 (Kinetoplastea; Trypanosomatidae), a hemoflagellate that infects more than 150 mammal species ^2^. Human transmission occurs mainly through contact with infected feces of triatomines of the genera *Triatoma*, *Rhodnius*, and *Panstrongylus*^3^ and through the ingestion of contaminated food. The diversity of reservoirs, hosts, and vectors has allowed for the mapping of its geographical distribution, genetic variability, and its association with the clinical manifestations of CD. Currently, *T. cruzi* is classified into seven discrete typing units (DTUs), TcI-TcVI and TcBat. Some of these DTUs are associated with cardiopathies, digestive megasyndromes, or indeterminate forms of CD ^1^.

In Colombia, the departments with the highest number of reported CD cases include Arauca, Boyacá, Casanare, Cundinamarca, Meta, Santander, Norte de Santander, Tolima, and Vichada. In the Caribbean region, the departments of Córdoba, Magdalena, La Guajira, Cesar, and Bolívar have concentrated most of the acute cases in recent years ^4^. In the department of Atlántico, the first imported case of CD was recorded in 2019. Furthermore, the presence of *P. geniculatus* (Latreille, 1811) and other triatomines has been documented in various municipalities, as well as the detection of *T. cruzi* in the municipality of Tubará^5^^,^^6^. However, the circulating genotypes in this region remain unknown. In this context, the objective of the present study was to identify the *T. cruzi* genotypes circulating in wild populations and those with a tendency toward domiciliation of *P. geniculatus* in three municipalities of the department of Atlántico, Colombia.

**KEY MESSAGES**

Study motivation. To determine the natural infection rate of *T. cruzi* in triatomines and identify the infecting *T. cruzi* genotypes, as this has not been investigated in the department of Atlántico.

Main findings. This is the first report of *P. geniculatus* naturally infected with the *T. cruzi* TcI genotype, and entomological indices for this species are reported for the first time in the department.

Public health implications. There is evidence of a high risk of infection for the inhabitants of the municipality of Tubará due to the presence of this vector infected with a pathogenic genotype in the domiciles and peridomiciles of the department of Atlántico.

## THE STUDY

A longitudinal descriptive study was conducted in rural areas characterized by a predominance of tropical dry forest in the municipalities of Piojó and Tubará, as well as in the urban periphery of the municipality of Usiacurí, department of Atlántico, Colombia.

In the municipality of Piojó, sampling was carried out in the Aguas Vivas district (323 m a.s.l.; 10°44’47.6”N, 75°06’08.8”W), while in the municipality of Tubará, it was conducted in the Juaruco district (200 m a.s.l.; 10°55’12.8”N, 74°59’30.4”W). Additionally, collections were made in the urban periphery of Usiacurí (95 m a.s.l.; 10°44’35.2”N, 74°58’21.1”W), a locality with average temperatures between 28 and 30 °C.

### Collection and processing of triatomine samples

Three samplings per study site were performed between March 2023 and May 2024, with an additional sampling in October 2025, for a total of 10 field trips and 30 sampling days. Triatomine collection was carried out through three complementary strategies: 1) active daytime and evening search inside dwellings in the peri-urban area of the municipality of Usiacurí, following the signing of informed consent and training of the head of the family or resident for triatomine identification; 2) in the municipalities of Piojó and Tubará, search in the peridomicile (30 m radius around the dwelling) using Shannon-type light traps, with checks every 30 min between 18:00 and 23:00 h, supplemented by active search in animal breeding sites, such as chicken coops and pens, during the same hours; and 3) in the forest area (≥30 m beyond the peridomicile), targeted search in palms, bird nests, wild animal burrows, holes, cracks, tree roots, and leaf litter. Collected specimens were transported to the molecular biology research laboratory of the Universidad del Atlántico (LIBM-UA) for identification. Using the taxonomic key of Lent and Wygodzinsky (1979) under a stereomicroscope, diagnostic characters, sex, and the size of the nymphal stage of the specimens were evaluated, followed by molecular and microbiological tests for the detection and typing of *T. cruzi*.

### DNA Extraction

Each collected specimen was initially subjected to ventral stimulation to obtain feces, followed by the collection of intestinal contents; samples were diluted 1:1 in saline solution (0.85%) and observed at 400X under a microscope. DNA extraction was performed following the instructions of the Wizard Genomic® purification kit from Promega ^7^.

### Detection of *T. cruzi* by polymerase chain reaction

For the identification of *T. cruzi* DNA, amplification of the variable region of the kinetoplast DNA (kDNA) minicircles was performed using primers S35 (5’-AAATAATGTACGGG(T/G) GAGATGCATGA3’) and S36 (5’GGGTTCGATTGGGGTTGGTGTAATATA-3’), which amplifies a 330 bp fragment ^(^^7^.

### Identification of the *T. cruzi* genotype (DTU) found in *P. geniculatus*

The DNA from kDNA-positive samples was used for genotyping through PCR amplification of the non-transcribed spacer regions of the mini-exon gene, using three primers: TC1 (5´-ACA CTT TCT GTG GCG CTG ATCG-3´); TC2 (5´-TTG CTC GCA CAC TCG GCT GCAT-3´); TC3 (5´-CCG CGW ACA ACC CCT MAT AAA AATG-3´) and the common primer ME (5´-TAC CAA TAT AGT ACA GAA ACTG-3´). Bands of 150, 200, or 250 bp indicate the detection of genotypes TcIII, TcI, or TcIV, respectively ^11^. Additionally, a region of the variable domain of the gene encoding the 18S rRNA was amplified using primers V1 (5´-CAA GCG GCT GGG TGG TTA TTC CA-3´) and V2 (5´-TTG AGG GAA GGC ATG ACA CAT GT-3´), for the detection of genotype TcI (175 bp) (TcII, TcIII, and TcV 165 bp) and TcIV (155 bp) ^7^^,^^8^. Electrophoresis gels were stained with SYBR Green (Thermo Fisher Scientific).

### Data Analysis

The obtained data were organized and analyzed using descriptive statistics, employing tables to describe the distribution of triatomines captured in the department of Atlántico, as well as the entomological indices and the presence of *T. cruzi*.

### Ethical Considerations

The informed consent for housing inspection and the triatomine collection protocol was evaluated and approved by evaluation act: No. 267 of May 26, 2022, by the Ethics Committee of the Universidad del Norte, Barranquilla, Colombia.

## FINDINGS

**Figure 1:**
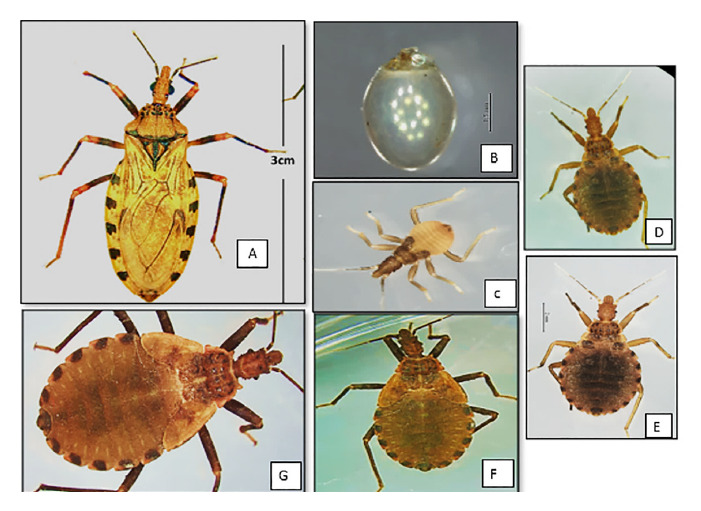
Specimens of *P. geniculatus* collected in three municipalities of the department of Atlántico. A) adult female, B) egg, C) 1st stage nymph, D) 2nd stage nymph, E) 3rd stage nymph. F) 4th stage nymph, G) 5th stage nymph.

**Figure 2:**
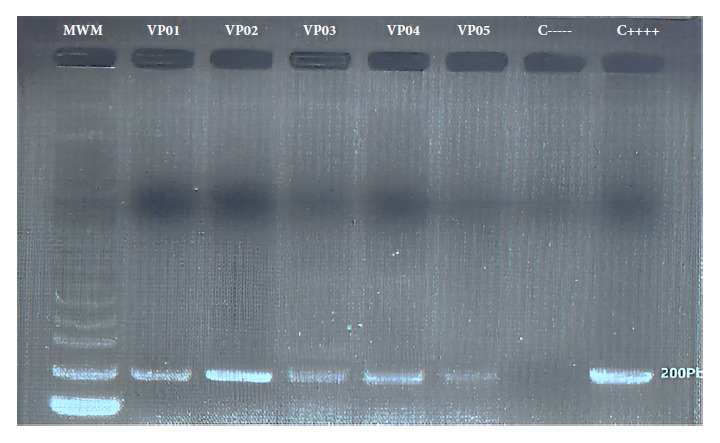
Identification of *T. cruzi*-TcI through amplification of the Mini-Exon gene in *P. geniculatus* collected in the municipality of Tubará.

**Figure 3:**
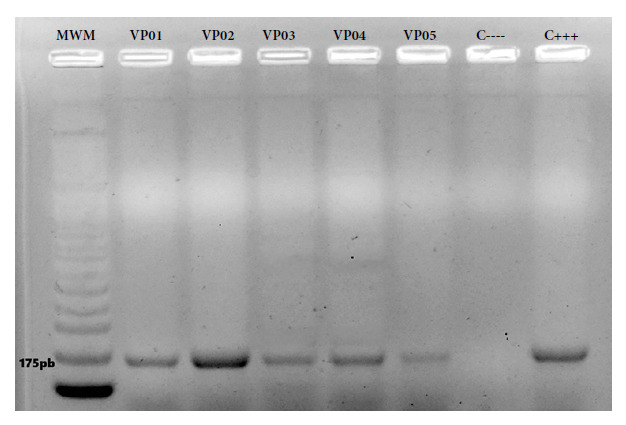
Identification of TcI through amplification of the 18S Ribosomal DNA gene in P. geniculatus collected in the municipality of Tubará.

A total of 35 specimens were collected, all belonging to the species *P. geniculatus* (Figure 1). Of these, two males were captured in dwellings in the peri-urban area of Usiacurí, in the intradomicile, around 19:00 hours during the month of April. In the municipality of Piojó, two females were collected in the peridomicile (a stone pigpen) of a rural dwelling at approximately 21:00 hours in September. Most triatomines (82.85%; 29/35), including adults, nymphs, and eggs, were collected in the municipality of Tubará. These were found mainly in the peridomicile, in the foundations of an abandoned house located less than 30 meters from an inhabited house, as well as on terraces of inhabited rural houses, whose structures were predominantly built of cement and covered with fiber-cement (Eternit®). Captures were made during the night and early morning in the months of March, May, and July (Table 1). 71.42% (25/35) of the total triatomines collected were found to be infected with *T. cruzi*, including several nymphal stages; 100% of the positives amplified for the TcI genotype (Figure 2 and Figure 3). It is important to highlight that all parasite-positive specimens were found in the municipality of Tubará.

Lanes (1) MWM: Molecular Weight Marker (100 bp). (2-6) VP01 female, VP02 female, VP03 5th stage nymph, VP04 male, VP05 male, (7) VP06 negative control of *P. geniculatus* feces free of *T. cruzi* infection and (8) VP08 positive control of triatomine positive for TcI lineage Venezuelan strain. 2.5% agarose gel and expected band of 200 bp for TcI lineage.

Lanes (1) MWM: Molecular Weight Marker (100 bp). (2-6) VP01 female, VP02 female, VP03 5th instar nymph, VP04 male, VP05 male, (7) C--- negative control of *P. geniculatus* feces free of *T. cruzi* infection, and (8) C+++ positive control of triatomine infected with *T. cruzi* genotype TcI from a Venezuelan strain. 2.5% agarose gel and expected band of 175 bp for TcI genotype.

**Table 1:** Distribution of *P. geniculatus* by stage, sex, and *T. cruzi* positivity in three municipalities of Atlántico.

Stage	Total	Usiacurí	Piojó	Tubará	
				Negative	Positive
Adults	20	2♂	2♀	1♀	11♀, 4♂
N5	3	_	_	—	2♂, 1♀
N4	3	_	_	—	3
N3	5	_	_	2	3
N2	2	_	_	1	1
N1	2	_	_	2	25
Total	35	2	2	6	25

N: nymphal stage, ♂ males, ♀ females.

### Entomological Indices

Triatomine infestation was identified in all three municipalities; however, entomological indices were only calculated for the municipality of Tubará because 82.85% of the specimens were captured there. The entomological indices seen in (table 2) show the high rate of infestation with the species *P. geniculatus* in houses, domiciles, and peridomiciles for the municipality of Tubará.

**Table 2:** Entomological indices for *P. geniculatus* from the domicile and peridomicile collected in the municipality of Tubará.

Entomological Indices	Index (%)
Housing infestation	60% (3/5)
Domiciliary infestations	60% (3/5)
Peridomiciliary infestation	40% (2/5)
Crowding^a^	9,6 (29/3)
Density^a^	5,8 (29/5)
Colonization	33% (1/3)
Housing infection	100% (3/3)
Natural infection of triatomines with *T. cruzi*	80% (25/31)

a The crowding and density indices were not expressed as percentages but as finite values greater than or equal to 1, and consisted of dividing the number of captured triatomines by the number of explored dwellings.

## DISCUSSION

*P. geniculatus* is a wild species considered a secondary vector of *T. cruzi* in Latin America; its presence is also recorded in peri-urban environments attracted by artificial light, and it has even been reported in domiciliation processes ^9^^,^^10^.

In Latin America, especially in the Amazon basin, oral transmission of *T. cruzi* has gained epidemiological relevance, associated with outbreaks due to contamination of food and beverages with feces from wild triatomines or secretions from infected reservoir mammals. In Colombia, oral transmission cases have been associated with *P. geniculatus* in areas of low endemicity ^11^, such as the department of Atlántico. Although this vector has been historically linked to wild cycles, its domiciliary presence gives it increasing epidemiological importance, particularly in the departments of Córdoba, Santander, and Meta ^12^.

In Atlántico, *P. geniculatus* was initially recorded in Piojó (2004) and subsequently in Luruaco, Puerto Colombia, Barranquilla, and Soledad (2012) ^5^; and in this study, Usiacurí. In 2015, a single specimen of *P. geniculatus* infected with *T. cruzi* collected in Tubará was reported in the department of Atlántico. The results of the present study demonstrate the collection of several triatomines of the species with a high rate of infection by *T. cruzi* of the TcI genotype (71.4 n:25/33), exceeding the country's average (71%) ^13^^,^^14^.

The high entomological index of domiciliary infestation observed confirms the frequent intrusion of adult specimens of *P. geniculatus* into the domicile. Likewise, the finding of eggs and various nymphal stages (N1-N5) inside an abandoned rural house, located approximately 30 m from an inhabited house, evidences a tendency toward domiciliation ^15^. This behavior is supported by a colonization index of 33.3%, which could be associated with the unplanned construction of housing in rural areas, the modification of the ecosystem, and the attraction of *P. geniculatus* toward artificial light sources both in the domicile and in peridomiciliary pens. It is worth noting that the houses where the insects were collected did not have mud or bahareque walls, nor structural conditions favorable for the establishment of triatomine colonies; however, the capture of specimens inside them was recorded. Additionally, the presence of triatomines positive for the TcI genotype in the three rural houses of the municipality of Tubará could indicate the existence of an active and fully established vector transmission cycle, which is why the infection rate in these houses was 100%.

The natural infection rate of 80% of *P. geniculatus* with the *T. cruzi* TcI genotype in the municipality of Tubará could be related to the capacity of this species, which has predominantly wild habits, to feed on various hosts, such as armadillos, rodents, opossums, and bats, from which it could have become infected with this genotype ^16^. These findings constitute a relevant element that reinforces the suspicion of its role as a vector in the wild transmission of *T. cruzi* in the department. In this context, it is fundamental to determine if this genotype also circulates in wild mammals, domestic animals, and humans in the municipality.

The presence of *P. geniculatus* in the domicile and peridomicile in the municipalities of interest in our research indicates a potential risk of transmission to humans, through possible contact with infected feces or food contamination with them, which could be associated with the epidemiological importance that *P. geniculatus* has been presenting in the oral transmission of CD in Colombia ^17^. In this context, it is recommended to investigate the presence of the parasite in domestic mammals and rodents to identify possible hosts and reservoirs in the department. Likewise, it is necessary to evaluate the vector's feeding sources to determine its potential hosts, as well as to conduct screenings in the human population. These actions will help guide the design of strategies for the prevention and control of *T. cruzi* transmission in the department of Atlántico.

As limitations, it should be mentioned that the low number of specimens captured in Piojó and Usiacurí could be related to the capture method employed; previous studies indicate a greater efficacy of traps baited with rodents or small birds compared to those used in this study ^18^^,^^19^. Likewise, this result could be associated with the limited number of samplings, so it is recommended that future entomological studies include a greater field effort and the use of traps baited with live animals.

In conclusion, this study reports for the first time the circulation of the *T. cruzi* TcI genotype in the department of Atlántico associated with *P. geniculatus*. A high infection rate is documented, with a domiciliation process in Tubará, and the geographical distribution is updated with the first record in the municipality of Usiacurí.
