# Supplementary material for: Circulation of Trypanosoma cruzi TcI in wild populations and with a tendency toward domiciliation of Panstrongylus geniculatus (Latreille, 1811) (Hemiptera; Reduviidae) in the department of Atlántico, Colombia
Source: Rev Peru Med Exp Salud Publica. 2026 Mar 6;43(1):83–9. doi: 10.17843/rpmesp.2026.431.15385 (PMC13245981; doi:10.17843/rpmesp.2026.431.15385)
Supplement: Translation

# Translation: Circulación de *Trypanosoma cruzi* TcI en poblaciones silvestres y con tendencia a la domiciliación de *Panstrongylus geniculatus* (Latreille, 1811) (Hemíptera; Reduviidae) en el departamento del Atlántico, Colombia

**Authors:** Yeisson Cera-Vallejo, Claudia Romero-Vivas, Andrew K. Falconar, Roberto García-Alzate, Daisy Lozano-Arias

**Affiliations:** 1 Grupo de investigación en Enfermedades Tropicales, Universidad del Norte, Puerto Colombia, Atlántico, Colombia.; 2 Grupo de investigación en Ciencias Básicas y Clínicas GIBAC, Facultad de Ciencias de la Salud, Fundación Universitaria San Martín-Puerto Colombia, Atlántico, Colombia.; 3 Grupo de investigación en Ciencia Educación y Tecnología CETIC, Universidad del Atlántico, Puerto Colombia, Atlántico Colombia.

**Keywords:** Trypanosoma cruzi, Genotipo, Panstrongylus

## Abstract

Se identificó el genotipo TcI de *Trypanosoma cruzi* en poblaciones silvestres y con tendencia a la domiciliación de *Panstrongylus geniculatus* en el departamento del Atlántico, Colombia. Mediante búsqueda activa y trampas de luz en zonas rurales y urbanas de tres municipios se capturaron 33 ejemplares de *P. geniculatus*: Usiacurí (2), Piojó (2) y Tubará (29), estos últimos recolectados en cinco viviendas. El ADN del contenido digestivo fue analizado por PCR empleando marcadores moleculares (DNAk, Mini-Exón, 18S, DNAr). Se evidenció infección por *T. cruzi* en el 75% de los especímenes (25/33), todos procedentes de Tubará, con identificación exclusiva del genotipo TcI. Adicionalmente, se calcularon índices entomológicos, con índices de infestación, colonización e infección natural del 60%, 33% y 86%, respectivamente. Estos hallazgos constituyen el primer reporte de *T. cruzi* TcI en el departamento del Atlántico y el primer registro de *P. geniculatus* en Usiacurí, lo que evidencia un riesgo potencial para la salud pública colombiana.

## INTRODUCCIÓN

La enfermedad de Chagas (ECh) es una zoonosis de trasmisión, principalmente vectorial, que afecta comunidades vulnerables de 21 países de América Latina. Se estima alrededor de siete millones de personas infectadas, con 30 000 casos y 12 000 muertes anualmente ^1^. Su agente etiológico es *Trypanosoma* (Schizotrypanum) *cruzi* Chagas 1909 (Kinetoplastea; Trypanosomatidae), un hemoflagelado que infecta a más de 150 especies de mamíferos ^2^. La transmisión humana ocurre principalmente por contacto con heces infectadas de triatominos de los géneros *Triatoma, Rhodnius y Panstrongylus*^3^ y por la ingestión de alimentos contaminados. La diversidad de reservorios, hospedadores y vectores ha permitido mapear su distribución geográfica, su variabilidad genética y su asociación con las manifestaciones clínicas de la ECh. Actualmente *T. cruzi* se clasifica en siete unidades discretas de tipificación (UDT), TcI-TcVI y TcBat. Algunas de estas UDT están asociadas con cardiopatías, megasíndromes digestivos o formas indeterminadas de la Ech ^1^. 

En Colombia, los departamentos con mayor número de casos reportados de ECh incluyen Arauca, Boyacá, Casanare, Cundinamarca, Meta, Santander, Norte de Santander, Tolima y Vichada. En la región Caribe, los departamentos de Córdoba, Magdalena, La Guajira, Cesar y Bolívar han concentrado la mayoría de los casos agudos en los últimos años ^4^. En el departamento del Atlántico, en 2019 se registró el primer caso importado de ECh. Además, se ha documentado la presencia de *P. geniculatus* (Latreille, 1811) y otros triatominos en diversos municipios, así como la detección de *T. cruzi* en el municipio de Tubará ^5^^,^^6^. Sin embargo, aún se desconocen los genotipos circulantes en esta región. En este contexto, el objetivo del presente estudio fue identificar los genotipos de *T. cruzi* que circulan en poblaciones silvestres y con tendencia a la domiciliación de P*. geniculatus* en tres municipios del departamento del Atlántico, Colombia.

**MENSAJE CLAVE**

Motivación del estudio. Conocer la tasa de infección natural por *T. cruzi* en triatominos e identificar los genotipos de *T. cruzi* infectantes, esto no ha sido investigado en el departamento del Atlántico.

Principales hallazgos. Este es el primer reporte de *P. geniculatus* infectado naturalmente con el genotipo TcI de *T. cruzi* y se reportan por primera vez los índices entomológicos para esta especie en el departamento.

Implicancia en salud pública. Se evidencia el alto riesgo de infección para los habitantes del municipio de Tubará por la presencia de este vector infectado con un genotipo patógeno en los domicilios y peridomicilio del departamento del Atlántico.

## EL ESTUDIO

Se realizó un estudio longitudinal descriptivo en áreas rurales caracterizadas por el predominio de bosque seco tropical, en los municipios de Piojó y Tubará, así como en la periferia urbana del municipio de Usiacurí, departamento del Atlántico, Colombia.

En el municipio de Piojó, el muestreo se llevó a cabo en la vereda Aguas Vivas (323 m.s.n.m.; 10°44’47.6”N, 75°06’08.8”W), mientras que en el municipio de Tubará se realizó en el corregimiento Juaruco (200 m.s.n.m.; 10°55’12.8”N, 74°59’30.4”W). Adicionalmente, se efectuaron colectas en la periferia urbana de Usiacurí (95 m.s.n.m.; 10°44’35.2”N, 74°58’21.1”W), localidad que presenta temperaturas promedio entre 28 y 30 °C.

### Recolección y procesamiento de muestras de triatominos

Se realizaron tres muestreos por sitio de estudio entre marzo de 2023 y mayo de 2024, con un muestro adicional en octubre de 2025, para un total de 10 salidas de campo y 30 días de muestreo. La colecta de triatominos se realizó mediante tres estrategias complementarias: 1) búsqueda activa diurna y vespertina en el interior de viviendas de la zona periurbana del municipio de Usiacurí, previa firma del consentimiento informado y capacitación al jefe de familia o residente para la identificación de triatominos; 2) en los municipios de Piojó y Tubará, búsqueda en el peridomicilio (radio de 30 m alrededor de la vivienda) mediante trampas de luz tipo Shannon, con revisiones cada 30 min entre las 18:00 y 23:00 h, complementada con búsqueda activa en sitios de cría de animales, como gallineros y corrales, en el mismo horario; y 3) en la zona de bosque (≥30 m más allá del peridomicilio), búsqueda dirigida en palmas, nidos de aves, madrigueras de animales silvestres, huecos, grietas, raíces de árboles y hojarasca. Los especímenes colectados fueron transportados al laboratorio de investigación en biología molecular de la Universidad del Atlántico (LIBM-UA) para su identificación, mediante la clave taxonómica de Lent y Wygodzinsky (1979), bajo un estereomicroscopio se evaluaron los caracteres diagnósticos, el sexo y el tamaño del estadio ninfal de los ejemplares, para posteriormente realizar pruebas moleculares y microbiológicas para la detección y tipificación de *T. cruzi*.

### Extracción de ADN

Cada uno de los especímenes colectados fueron sometidos inicialmente a estimulación ventral para obtención de heces, seguido de obtención del contenido intestinal; las muestras fueron diluidas 1:1 en solución salina (0,85%) y observadas a 400X bajo microscopio. La extracción de ADN se realizó siguiendo las instrucciones del kit de purificación Wizard Genomic® de Promega ^7^.

### Detección de *T. cruzi* por reacción en cadena de la polimerasa

Para la identificación de ADN de *T. cruzi* se realizó la amplificación de la región variable de los minicírculos del ADN del kinetoplasto (ADNk) usando los iniciadores S35 (5’-AAATAATGTACGGG(T/G) GAGATGCATGA3’) y S36 (5’GGGTTCGATTGGGGTTGGTGTAATATA-3’), que amplifica un fragmento de 330 pb ^7^. 

### Identificación del genotipo (DTU) de *T. cruzi* encontradas en *P. geniculatus*

El ADN de las muestras positivas para ADNk fue usado para genotipificar mediante la amplificación por PCR de las regiones del espaciador no transcrito del gen del mini-exón, usando tres iniciadores TC1 (5´-ACA CTT TCT GTG GCG CTG ATCG-3´); TC2 (5´-TTG CTC GCA CAC TCG GCT GCAT-3´) y TC3 (5´-CCG CGW ACA ACC CCT MAT AAA AATG-3´) y el iniciador común ME (5´-TAC CAA TAT AGT ACA GAA ACTG-3´). Las bandas de 150, 200 o 250 pb indican la detección de los genotipos TcIII, TcI o TcIV, respectivamente ^11^. Adicionalmente se amplificó una región del dominio variable del gen que codifica el ARNr 18S, usando los iniciadores V1 (5´-CAA GCG GCT GGG TGG TTA TTC CA-3´) V2 (5´-TTG AGG GAA GGC ATG ACA CAT GT-3´), para la detección del genotipo TcI (175 pb) (TcII, TcIII y TcV 165 pb) y TcIV (155pb) ^7^^,^^8^. Los geles para electroforesis fueron teñidos con SYBR Green (Thermo Fisher Scientific).

### Análisis de datos

Los datos obtenidos fueron organizados y analizados mediante estadística descriptiva, utilizando tablas para describir la distribución de los triatominos capturados en el departamento del Atlántico, así como, los índices entomológicos y la presencia de *T. cruzi*.

### Consideraciones éticas

El consentimiento informado para la inspección de viviendas y el protocolo de recolección de triatominos fue evaluado y aprobado mediante el acta de evaluación: N.º 267 del 26 de mayo de 2022 por el Comité de Ética de la Universidad del Norte, Barranquilla, Colombia.

## HALLAZGOS

Se colectaron 35 especímenes, todos pertenecientes a la especie *P. geniculatus* (figura 1). De estos, dos machos fueron capturados en viviendas del área periurbana de Usiacurí, en el intradomicilio, alrededor de las 19:00 horas durante el mes de abril. En el municipio de Piojó, se recolectaron dos hembras en el peridomicilio (corral de piedra para cerdos) de una vivienda rural, aproximadamente a las 21:00 horas en septiembre. La mayoría de los triatominos (82,85%; 29/35), incluyendo adultos, ninfas y huevos, se colectaron en el municipio de Tubará. Estos fueron encontrados principalmente en el peridomicilio, en los cimientos de una vivienda abandonada ubicada a menos de 30 metros de una vivienda habitada, así como en terrazas de viviendas rurales habitadas, cuyas estructuras estaban predominantemente construidas en cemento y cubiertas con fibrocemento (Eternit®). Las capturas se realizaron durante la noche y la madrugada, en los meses de marzo, mayo y julio (tabla 1). El 71,42% (25/35) del total de los triatominos colectados, se encontraron infectados con *T. cruzi*, incluyendo varios estadios ninfales*,* el 100% de los positivos amplificaron para genotipo TcI (figura 2 y figura 3). Es importante destacar que todos los ejemplares positivos al parásito fueron encontrados en el municipio de Tubará.

**Figura 1:** Ejemplares colectados de *P. geniculatus* en tres municipios del departamento del Atlántico. A) hembra adulta, B) huevo, C) ninfa estadio 1, D) ninfa estadio 2, E) ninfa estadio 3. F) ninfa estadio 4, G) ninfa estadio 5.

**Tabla 1:** Distribución de *P. geniculatus* por estadio, sexo y positividad para *T. cruzi* en tres municipios del Atlántico.

Estadio	Total	Usiacurí	Piojó	Tubará	
				Negativos	Positivos
Adultos	20	2♂	2♀	1♀	11♀, 4♂
N5	3	_	_	-	2♂, 1♀
N4	3	_	_	-	3
N3	5	_	_	2	3
N2	2	_	_	1	1
N1	2	_	_	2	25
Total	35	2	2	6	25

N: estadio ninfal, ♂ machos, ♀ hembras.

**Figura 2:** Identificacion de *T. cruzi-*TcI, mediante amplificacion del gen del Mini-Exon en *P. geniculatus* colectados en el municipio de Tubara.

Pozos (1) MPM: Marcador de Peso Molecular (100 pb). (2-6) VP01 hembra, VP02 hembra, VP03 Ninfa estadio 5, VP04 Macho, VP05 Macho, (7) VP06 control negativo de heces de *P. geniculatus* libre de infeccion con *T. cruzi* y (8) VP08 control positivo de triatomino positivo a linaje TcI cepa venezolana. Gel al 2,5% de agarosa y banda esperada de 200 pb para linaje TcI.

**Figura 3:** Identificacion de TcI mediante la amplificacion del gen 18S ADN Ribosomal en *P. geniculatus* colectados en el municipio de Tubara.

Pozos (1) MPM: Marcador de Peso Molecular (100 pb). (2-6) VP01 hembra, VP02 hembra, VP03 Ninfa estadio 5, VP04 Macho, VP05 Macho, (7) C--- control negativo de heces de *P. geniculatus* libre de infeccion con *T. cruzi* y (8) C+++ control positivo de triatomino infectado con *T. cruzi* genotipo TcI de una cepa venezolana. Gel al 2,5 % de agarosa y banda esperada 175 pb para genotipo TcI.

### Índices entomológicos

En los tres municipios se identificó infestación por triatominos, sin embargo, solo se calcularon los índices entomológicos para el municipio de Tubará debido a que el 82,85% de los ejemplares fueron capturados en este municipio, los índices entomológicos que se aprecian en la (tabla 2) muestran la alta tasa de infestación con la especie *P. geniculatus* en viviendas, domicilio y peridomicilio para el municipio de Tubará.

**Tabla 2:** Índices entomológicos para *P. geniculatus* del domicilio y peridomicilio recolectados en el municipio de Tubará.

Índices entomológicos	Índice (%)
Infestación de vivienda	60% (3/5)
Infestación del domicilio	60% (3/5)
Infestación del peridomicilio	40% (2/5)
Hacinamiento^a^	9,6 (29/3)
Densidad^a^	5,8 (29/5)
Colonización	33% (1/3)
Infección de viviendas	100% (3/3)
Infección natural de triatominos con *T. cruzi*	80% (25/31)

a Los índices de hacinamiento y densidad no fueron expresados como porcentajes sino como valores finitos mayores o iguales a 1, y consistió en dividir el número triatominos capturados sobre el número de viviendas exploradas.

## DISCUSIÓN

*P. geniculatus* es una especie silvestre considerada vector secundario de *T. cruzi* en América Latina, también registra su presencia en ambientes periurbanos atraídos por luz artificial e incluso se ha reportado en procesos de domiciliación ^9^^,^^10^.

En América Latina, especialmente en la cuenca amazónica, la transmisión oral de *T. cruzi* ha ganado relevancia epidemiológica, asociada a brotes por contaminación de alimentos y bebidas con heces de triatominos silvestres o secreciones de mamíferos reservorios infectados. En Colombia, los casos de transmisión oral se han asociados a *P. geniculatus* en zonas de baja endemicidad ^11^, como el departamento del Atlántico. Aunque este vector ha estado históricamente ligado a ciclos silvestres, su presencia domiciliaria le confiere creciente importancia epidemiológica, particularmente en los departamentos de Córdoba, Santander y Meta ^12^.

En el Atlántico, *P. geniculatus* fue registrado inicialmente en Piojó (2004) y. posteriormente. en Luruaco, Puerto Colombia, Barranquilla y Soledad (2012) ^5^ y en este estudio Usiacurí, en 2015, se reporta en el departamento del Atlántico en un único ejemplar de *P. geniculatus* infectado *con T. cruzi* colectado en Tubará. Los resultados del presente estudio evidencian la colecta de varios triatominos de la especie con una alta tasa de infección por *T. cruzi* del genotipo TcI. (71.4 n:25/33), superando el promedio del país (71%) ^13^^,^^14^.

El alto índice entomológico de infestación domiciliaria observado confirma la intrusión frecuente de especímenes adultos de *P. geniculatus* en el domicilio. Asimismo, el hallazgo de huevos y distintos estadios ninfales (N1-N5) en el interior de una vivienda rural abandonada, localizada a aproximadamente 30 m de una casa habitada, evidencia una tendencia a la domiciliación ^15^. Este comportamiento se ve respaldado por un índice de colonización del 33,3%, el cual podría estar asociado a la construcción no planificada de viviendas en áreas rurales, la modificación del ecosistema y la atracción de *P. geniculatus* hacia fuentes de luz artificial tanto en el domicilio como en corrales del peridomicilio. Cabe resaltar que las viviendas donde se colectaron los insectos no presentaban paredes de barro o bahareque, ni condiciones estructurales favorables para el establecimiento de colonias de triatominos; sin embargo, se registró la captura de ejemplares en su interior. Adicionalmente, la presencia de triatominos positivos al genotipo TcI en las tres viviendas rurales del municipio de Tubará podría indicar la existencia de un ciclo de transmisión vectorial activo y plenamente establecido, razón por la cual el índice de infección en estas viviendas fue del 100%.

El índice de infección natural del 80% de *P. geniculatus* con el genotipo TcI de *T. cruzi* en el municipio de Tubará podría estar relacionado con la capacidad de esta especie, de hábitos predominantemente silvestres, para alimentarse de diversos hospedadores, como armadillos, roedores, zarigüeyas y quirópteros, en los cuales pudo haberse infectado con este genotipo ^16^. Estos hallazgos constituyen un elemento relevante que refuerza la sospecha de su papel como vector en la transmisión silvestre de *T. cruzi* en el departamento. En este contexto, resulta fundamental determinar si este genotipo también circula en mamíferos silvestres, domésticos y en humanos en el municipio.

La presencia de *P. geniculatus* en el domicilio y peridomicilio, en los municipios de interés en nuestra investigación, indican un potencial riesgo de transmisión para los humanos, por posible contacto con las heces infectadas o por contaminación de los alimentos con estas, lo que podría estar asociado con la importancia epidemiológica que ha venido presentando *P. geniculatus* en la transmisión oral de ECh en Colombia ^17^. En este contexto, se recomienda investigar la presencia del parásito en mamíferos domésticos y roedores, con el fin de identificar posibles hospedadores y reservorios en el departamento. Asimismo, es necesario evaluar las fuentes de alimentación del vector para determinar sus hospedadores potenciales, así como realizar tamizajes en la población humana. Estas acciones permitirán orientar el diseño de estrategias de prevención y control de la transmisión de *T. cruzi* en el departamento del Atlántico.

Como limitaciones se debe mencionar que el bajo número de especímenes capturados en Piojó y Usiacurí podría estar relacionado con el método de captura empleado, estudios previos indican una mayor eficacia de trampas cebadas con roedores o aves pequeñas frente a las utilizadas en este estudio ^18^^,^^19^. Asimismo, este resultado podría asociarse al limitado número de muestreos, por lo que se recomienda que futuros estudios entomológicos incluyan un mayor esfuerzo de campo y el uso de trampas cebadas con animales vivos.

En conclusión, este estudio reporta por primera vez la circulación del genotipo TcI de *T. cruzi* en el departamento del Atlántico asociada a *P. geniculatus.* Se documenta una alta tasa de infección, con un proceso de domiciliación en Tubará y se actualiza la distribución geográfica con el primer registro en el municipio de Usiacurí.
